# The effect of type of specialty (periodontology/oral surgery) on early implant failure: a retrospective “Big-Data” study from a nation-wide dental chain in Israel

**DOI:** 10.1007/s00784-022-04565-z

**Published:** 2022-06-27

**Authors:** Findler Mordechai, Chackartchi Tali, Mann Jonathan, Platner Ori, Bernstein Yaron, Shefer Ram, Tobias Guy

**Affiliations:** 1 Dental Research Unit – Maccabi-Dent, Maccabi Healthcare Fund, Tel Aviv, Israel; 2 Department of Periodontology, Hadassah Medical Center, Faculty of Dental Medicine, Hebrew University of Jerusalem, Jerusalem, Israel; 3 Oral Medicine Unit, Sheba Medical Center, Tel-Hashomer, Ramat Gan, Israel; 4 Department of Community Dentistry, Hadassah Medical Center, Faculty of Dental Medicine, Hebrew University of Jerusalem, Jerusalem, Israel

**Keywords:** Dental implant failure, Nested case-case, *Big Database Research*, Periodontists, Oral surgeons, Early implant failure

## Abstract

**Objectives:**

To evaluate early implant failure rate of implants placed by maxillofacial-oral surgeons and periodontists.

**Material and methods:**

A nested case-case study was performed to analyze treatment outcome of 27 oral surgeons and 30 periodontists who performed at least 100 dental implants between 2017 and 2019 in 54 clinics of “Maccabi-Dent,” a nation-wide dental chain. A total of 26,865 implants were evaluated.

**Results:**

The early failure rate of 1.3% achieved by the periodontists was lower than the 1.7% early failure rate achieved by oral surgeons. Differences were not statistically different. Oral surgeons in the study cohort were insignificantly older in age, with more years of experience as dentists and as specialists. However, the only parameter found to be a predictor to early implant failure in a linear regression model was related to postgraduate training. Explicitly, the mean number of implants placed during specialty program. This number was higher for the periodontists and found to be significantly contributing predictor to early implant failure. Clinicians’ age and years of experience as dentists or as specialist were not found to be predictors to early implant failure rate.

**Conclusions:**

No statistically significant differences were found in early implant failure rate between oral surgeons and periodontists. The number of implants placed during specialty program has a statistical predictive value to early implant failure rate.

**Clinical relevance:**

Care and attention should be taken to re-evaluate clinical training in the field of implantology during specialty program. To optimize surgeons’ control on treatment outcome.

## Introduction

Early implant failure is defined as failure prior to the connection of prosthetic restoration [1] and considered to be low [2]. Early implant failure is usually attributed to biological factors, surgical trauma, and impaired healing that resulted in a failure to achieve osseointegration [3]. However, the success of dental implants is multifactorial, including the experience and expertise of the treatment provider. Possible correlation between inexperience and higher failure rates was indicated already in an early work by Brânemark et al. [4]. The professional training and implant-related knowledge applied by practitioners is diverse [5]. It was suggested that the dentist’s years of experience, implant training, and postgraduate specialization might affect the knowledge, attitude, and way of practice of dental implants [5]. Sonkar et al. [6] reported a higher survival rate of 94.14% for residents in periodontology, followed by residents in prosthodontists (91.48%) and residents in maxillofacial oral surgeons (89.64%). The survival rates of implants improved by year of training: third year periodontics and oral surgeons 94.2%, second year 89.38%, and first year 88.6%. Nevertheless, only few studies have been published comparing dental implant treatment outcome based on the training of the surgeon, specifically comparing periodontists (PR) and maxillofacial oral surgeons (OS).

In order to address the scarcity of data regarding the contribution of the clinician as a factor influencing implant success, we studied early implant failure of implants inserted by specialists in periodontology (PR) and specialists in maxillofacial-oral surgery (OS), focusing on the effects of seniority as well as the type of care giver training on the early success rate of the procedure.

## Methods

A retrospective study was performed. Based on the dental records of patients treated by 57 dental professionals between 2017 and 2019 at “Maccabi-Dent,” the dental branch of “Maccabi,” the second largest Health Management Organization (HMO) in Israel. The inclusion criteria of clinicians participating in the study comprised of the following: (1) clinicians who are board-certified specialists that had finalized an accredited specialty program and were certified by the Israeli Ministry of Health. (2) All the involved clinicians performed more than 100 implants in “Maccabi-Dent” dental chain clinics throughout the observation period.

Data collection regarding the treating surgeon included the following: type of specialty (P/OS), age, DMD seniority (years of experience as a dentist), seniority as a specialist (years of experience as a specialist), number of implants placed during the specialty program. This information was obtained by personal inquiry of each participating surgeon.

Implant-related data was obtained from the crude data supplied by “Maccabi-Dent” big data database center. In the database, patient records are computerized and coded according to the performed procedure. The accuracy of the data is obtained from the method of payment to the doctors. The treating doctor will report the procedures performed by codes to be payed accordingly. Additional daily administrative supervision is constantly conducted, at the level of the clinic, validating the accuracy of reported codes.

To support the null hypothesis, a specific data-extracting algorithm was designed and implant-related records were obtained according to it. Inclusion criteria included all implants performed by the included specialists, were removed/extracted after up to 12 months following implant insertion, and had complete dental records available in the database. Dental records included information regarding the surgical procedure and follow-up meetings, up to 1 year following implant placement. Incomplete records, or records with clear errors, were excluded. Additional procedures conducted simultaneous to implant placement were also recorded, including simultaneous maxillary sinus augmentation, simultaneous horizontal bone augmentation, and post extraction immediate implant placement (i.e., complex procedures).

### Statistical methods

Records were extracted to an excel sheet. Dependent variable was implant failure rate after 1 year. Data were analyzed with IBM SPSS statistics software version 27.0. The significance levels were set at 0.05. Baseline characteristics are presented as minimum value, maximum value, means and standard deviations for continuous variables, and frequencies and percentages for categorical variables for the dependent variables. Independent *T*-tests were conducted to find correlation with specialty type and procedures. Pearson correlations were performed to find connections between dependent and independent variables and multiple logistic regression (Enter) tests were applied on control predictive variables (age, dentist seniority, dental specialist seniority, and the addition of a complex procedure to implant placement).

## Results

The study was approved by the local ethical committee at Maccabi Health Institute-IRB (ASMC-0032–20). A total of 57 surgeons participated in this study: 27 (47%) certified oral surgeons (OS) and 30 (53%) periodontists (PR). OS (M = 49.18, SD = 5.58) in the study group are older than PR (M = 46.52, SD = 7.35); differences are not statistically significant (Table 1). OS in the study group have longer experience as dentists (time passed from completion of DMD training) (M = 21.58, SD = 8.73) and have longer experience as experts (expert seniority) (M = 13.54, SD = 7.11) comparing to PR (mean duration from completion of DMD: M = 17.37, SD = 7.18) (mean duration as experts: M = 9.13, SD = 5.98). However, these differences are not statistically significant (Table 1). PR placed an average of 128.9 implants during their specialty program, compared to an average of 31.67 implants placed by OS (Table 1).

**Table 1:** Surgeons’ characteristics, number of implants placed and failure rate per practitioner

	Oral surgeons		*Periodontists*		Difference		
Variable	M	SD	M	SD	*T*(d.f)	d.f	*p*
Age	49.18	5.58	46.52	7.35	1.401	47	.084
DMD seniority	21.58	8.73	17.37	7.18	1.615	40	.057
Expert seniority	13.54	7.11	9.13	5.97	2.096	41	.021
Number of implants during internship	31.67	31.67	128.99	61.81	− 6.240	37	< .001
Rate of failures	0.017	0.017	0.013	.01	1.418	35	0.083

A total of 26,865 implants were placed by the cohort of clinicians during the observation time (between 2017 and 2019). 91.7% of the implants were manufactured by MIS Dental Implants company, 6.9% were by Alpha-Bio Tec. Dental Implants, 1.04% by Zimmer Biomet Dental, and 0.2% by Adin Dental Implant Systems Ltd.

Forty-five percent of these implants were placed by OS, of which 220 failed in the first year (1.7%). PR placed 55% of the total amount of implants, of which 170 (1.3%) failed and were removed in the first year (Table 1). The number of failed implants was divided by the total number of implants placed by the clinician and was expressed as early failure rate (Table 1). No significant differences were found between the two groups (OS/PR) regarding the total number of inserted implants and the mean early failure rate.

A total of 1302 (2.55%) implants were installed simultaneous with an additional procedure (immediate to tooth extraction/with a simultaneous crestal bone augmentation/with a simultaneous bone augmentation). OS (M = 0.88, SD = 2.21) did less procedures including simultaneous bone augmentation (Table 2). This was significantly significant. No other significant differences were found in the comparison of the use of additional complex procedures between the two types of specialists (OS/PR) (Table 2).

**Table 2:** Number of additional procedures performed simultaneous to implant placement. (Immediate implant/sinus augmentation/horizontal bone augmentation)

	Oral surgeons		*Periodontists*		Difference		
Variable	M	SD	M	SD	*T*(d.f)	d.f	*p*
Immediate implants	16.13	19.44	25.44	50.72	-.846	49	.201
Sinus augmentation	1.01	1.50	1.63	3.73	-.772	49	.222
Horizontal bone augmentation	0.88	2.21	5.15	12.44	− 1.754	49	.045

**Fig. 1:**
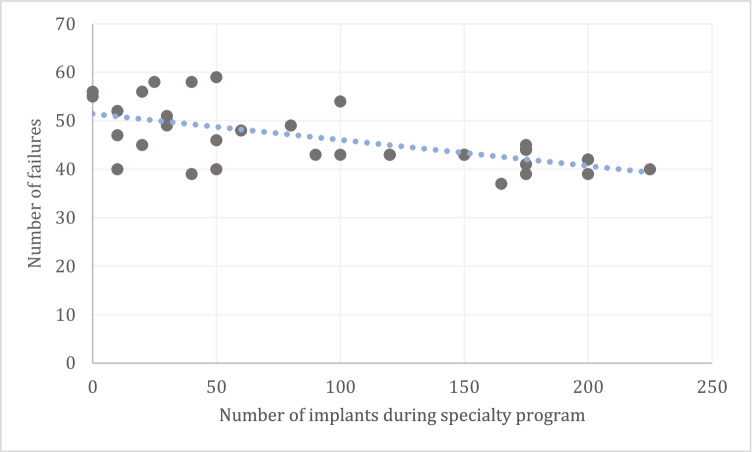
Number of failed implants during the study—according to the number of implants placed during the clinicians’ specialty program

Additionally, a multiple linear stepwise regression model was formed considering the following predictors: specialty type (OS/PR), DMD seniority, expert seniority, number of implant procedures during specialty training, immediate implantation, sinus augmentation, and horizontal bone augmentation to predict early failure rates. The whole model was found to be significant with an explained variance percentage of 14.8% {*F* (1.25) = 4.349, *p* = 0.47}. The predictor that significantly contributed to the model was “type of specialty” (OS/PR) (*β* =  − 0.385, *p* = 0.047); and when used, there was a significant increase in early implant failure (Fig. 1). Pearson test for evaluation of the correlation between number of early implant failures and the different variables revealed the number of implant procedures performed during specialty training to be a predictor for early implant failure. For example, the more procedures the surgeon did during specialty training, the less early implant failure they exhibited in the duration of the study (*r* =  − 0.352, *p* = 0.014) (Fig. 1). Pearson test implemented on each cohort separately (OS/PR) revealed in the group of periodontists a significant negative correlation between a simultaneous sinus augmentation and early implant failure rate (*r* =  − 0.381, *p* = 0.040). For example, periodontists preforming more simultaneous bone augmentation exhibited a lower early failure rate during the study. Clinicians’ age and years of experience as dentists or as specialist were not found to be predictors to early implant failure rate.

## Discussion

Dental implants are an effective and predictable treatment modality for replacing missing teeth in fully and partially edentulous patients. Nevertheless, despite high implant survival and success rates, failure might occur at an early or a late stage. Early implant failure is usually attributed to a biological complication [3, 7]. Anatomical conditions, such as bone quality, and patient-related systemic conditions, such as smoking and diabetes, are often discussed [8]. However, the scientific literature is lacking reports on the possible influence of the surgeons’ dental/implant-related education and its relevance to treatment outcome. This study investigates early failure rate of dental implants placed by certified specialists in periodontology and maxillofacial-oral surgery at “Maccabi-Dent” dental chain clinics in Israel. PR were found to have less early implant failure rate comparing to OS. Differences were not statistically significant. However, the number of implants placed during the postgraduate training was found to be significantly predicting early failure rate of implants placed years after training. In the cohort of clinicians participating in the study, the periodontists had placed more implants during their postgraduate specialty program. The number of implants placed during the specialty program was found to be a predictor to the early failure rate of implants, despite long years of experience and high number of implants placed after postgraduate training. These results imply considerable influence of the initial training of the clinician on treatment outcome in the years to come. These results are in contrast to a study by Melo et al. [9] who investigated the influence of the level of oral and maxillofacial resident training on the outcome of dental implant survival rates. This study included 175 implants placed in 54 patients. The overall survival rate of implants placed by residents at all levels of training was 91%. No statistically significant difference in implant survival rates was observed as a function of the level of training of the resident surgeon (*p* = 0.89) or location of implant placement (*p* = 0.93) [9]. However, in this study, the number of implants placed by OS during their training was similar to the described for the PR group in our study. Sendyk et al. reviewed the evidence regarding the correlation between the expertise of surgeons and the survival rate of dental implants. The data from the included publications in this review suggest that surgical experience did not significantly affect implant failure when considering experience based on specialty, but were significantly affected when considering experience based on the number of implants placed.^10^ It is well established that surgical experience will positively affect early implant success rate [10]. Zoghbi et al. [1] looked at the influence of surgical experience on osseointegration of two-stage implant placement (265 implants were inserted in 110 patients). The group concluded that surgical experience acquired during and after a postgraduate program in “implant dentistry” appears to influence osseointegration of implants, with a higher osseointegration rate found in implants performed by more experienced professionals. For the first 50 implants (placed during the training program), the osseointegration rate was 84.0%, whereas in the implants performed after the program, the rate reached 94.4%. Similarly, in a comprehensive review by Jerjes and Hopper [11], the evidence clearly indicated that the surgeon’s experience positively correlates with the level of osseointegration and implant success [11]. This review reflects an agreement that trainees and less experienced surgeons take more time to undertake a procedure which, in theory, can delay tissue recovery and compromise outcome. In the current study, all clinicians were specialists with vast experience, therefore, as expected, exhibiting low rate of early failures. The mean time following specialty training was 22 years for OS and 18 for PR; this might blur the effect of uneven experience gained during training. However, due to the big number of implants scanned in the current study, we were able to detect the predictive value of number of implants placed during specialty training on early implant failure rate. The results presented in the current study describe a cohort of experienced state-accredited specialists (OS and PR) in Israel. As far as we know, this is the first statistical evidence for a correlation between the type of training and failure rate, despite years of clinical experience.

Pearson test of each group alone was able to detect a trend in the cohort of periodontists. There was a significant negative correlation between a simultaneous sinus augmentation and early implant failure rate. Several studies support the fact that simultaneous sinus augmentation will not impair implant survival rate [12, 13]. This correlation might imply that a trained specialist will feel confident to perform a more “complex” procedure. However, being skilled will lower failure rate.

The database for this study was derived from electronic medical records of patients treated by specialists (PR, OS) working in a nation-wide dental chain in Israel. In this dental chain clinics, only certified specialists are allowed to perform implant surgery; consequently, the study was evaluating a cohort of specialists with a certified 3–4-year state-accredited postgraduate training lacking the ability to extend it to additional study groups (e.g., general practitioners). This might include an inherent group selection bias since many practitioners installing implants around the world are trained in a shorter and more limited implant training programs. The scanned clinical activity was limited to Israel, therefore, representing a specific population. However, data from 54 clinics was evaluated. These clinics serve wide population including different age groups, ethnic origin, and socioeconomic status. The study encircles a total of 26,865 implants, installed by 47 clinicians working in 54 clinics. The big sample size might compensate for the abovementioned study limitations, representing “real-life” setting.

Further retrospective analysis of large databases might shed light on possible variables influencing treatment outcome in implant dentistry, thus should be a target for further research.

## Conclusion

Different academic training, specifically specialties in maxillofacial oral surgery or periodontology, are not expected to influence early implant failure rate, especially after several years of clinical experience. However, in a retrospective study including large number of implants, we could find a statistical predictive value to the number of implants placed during postgraduate training and early implant failure rate during the following years of practice.
